# Secondary ileal lymph node metastases from rectal cancer: a case report

**DOI:** 10.1186/s40792-024-01912-y

**Published:** 2024-05-11

**Authors:** Makoto Ando, Nobuyuki Takemura, Ryo Oikawa, Yuhi Yoshizaki, Fuyuki Inagaki, Fuminori Mihara, Tomomichi Kiyomatsu, Kazuhiko Yamada, Norihiro Kokudo

**Affiliations:** Hepato-Biliary-Pancreatic Surgery Division, Department of Surgery, National Center for Global Health and Medicine, 1-21-1 Toyama, Shinjuku-Ku, Tokyo, 162-8655 Japan

**Keywords:** Locally invasive rectal cancer, Secondary lymph node metastasis, Ileal lymph node metastasis

## Abstract

**Background:**

Colorectal cancer can invade adjacent organs, but rarely metastasizes to the regional lymph nodes (LNs) of the invaded organ. Herein, we report a case of rectal cancer invading the ileum and metastasized to the regional ileal LNs.

**Case presentation:**

A 77-year-old male presented abdominal pain and anorexia, diagnosed with rectal cancer invading the small intestine and concurrently metastasized to the regional LN of the intestine and liver. High anterior resection and partial resection of the small intestine was performed, then, the patient was referred to our hospital for chemotherapy. We performed 17 cycles of systemic chemotherapy that achieved a partial reduction in size of the LN, followed by an ileocecal resection with ileal mesentery resection for regional LNs removal. Histopathological analysis of the resected ileal LNs and six liver lesions revealed a moderately differentiated tubular adenocarcinoma. The patient was discharged on postoperative day 18. Cancer recurrences developed in the lungs 5 months after the surgery, then to the liver and peritoneum, and further surgery and chemotherapy were performed. Despite the challenging presentation, the patient survived for 40 months after the first surgery.

**Conclusions:**

We report a rare case of a surgical resection of a secondary ileal LN metastasis from rectal cancer. The patient survives for a relatively long time after surgical resection. When colorectal cancer invades the small intestine, clinicians should consider the possibility of secondary LN metastasis in the invaded site.

## Background

Approximately 10% of colorectal cancers invade adjacent organs, such as the small intestine, ovaries, and uterus [1, 2]. Therefore, radical resection concomitant with organ invasion is necessary in such cases. Radical resection is expected to result in a good prognosis for these patients. However, it is extremely rare for colorectal cancer to directly invade the adjacent small intestine and metastasize to the regional lymph nodes (LNs) of the invaded intestine. Such metastases, considered distant metastases, are rarely resected and their prognosis is unclear. Herein, we present a case of colon cancer with ileal invasion and ileal LN metastasis and a literature review of previous reports of similar cases.

## Case presentation

**Fig. 1:**
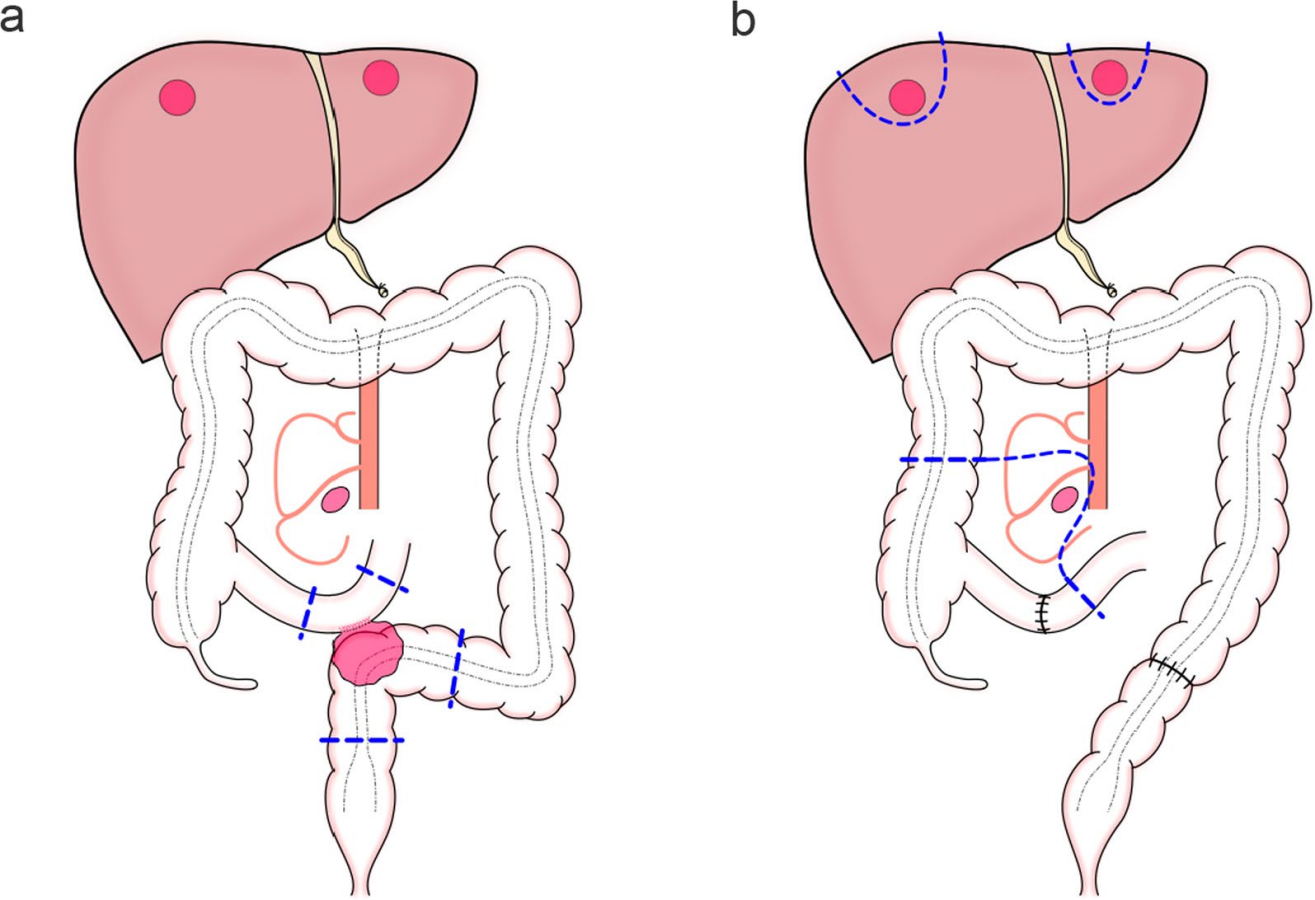
The schematic drawings of the surgery. **a** High anterior resection with D3 lymph node resection and partial resection of the small intestine is performed in the previous hospital. **b** Open ileocecal resection with ileal mesentery resection and multiple partial liver resections are performed in our hospital

**Fig. 2:**
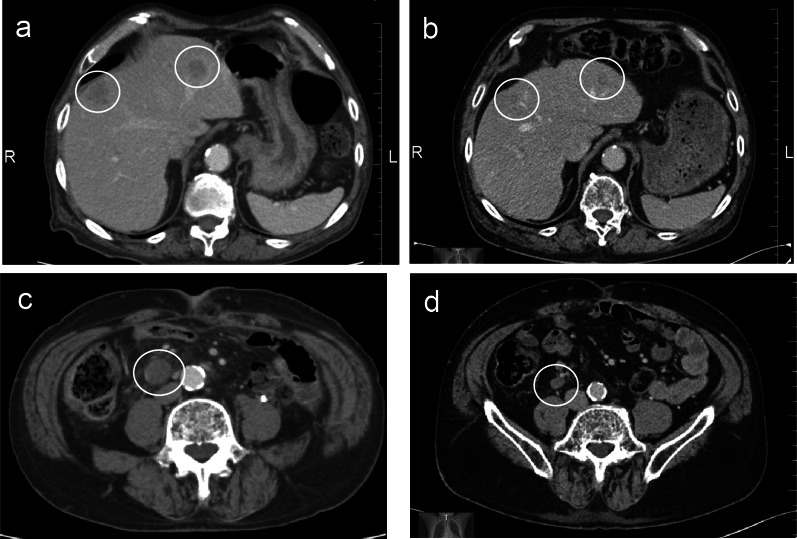
Change in tumor size. The size of the liver lesions decreased from **a** 27 mm and 17 mm to **b** 12 mm and 10 mm. The size of the ileal lymph node lesion decreased from the diameter of **c** 20 mm to **d** 12 mm

**Fig. 3:**
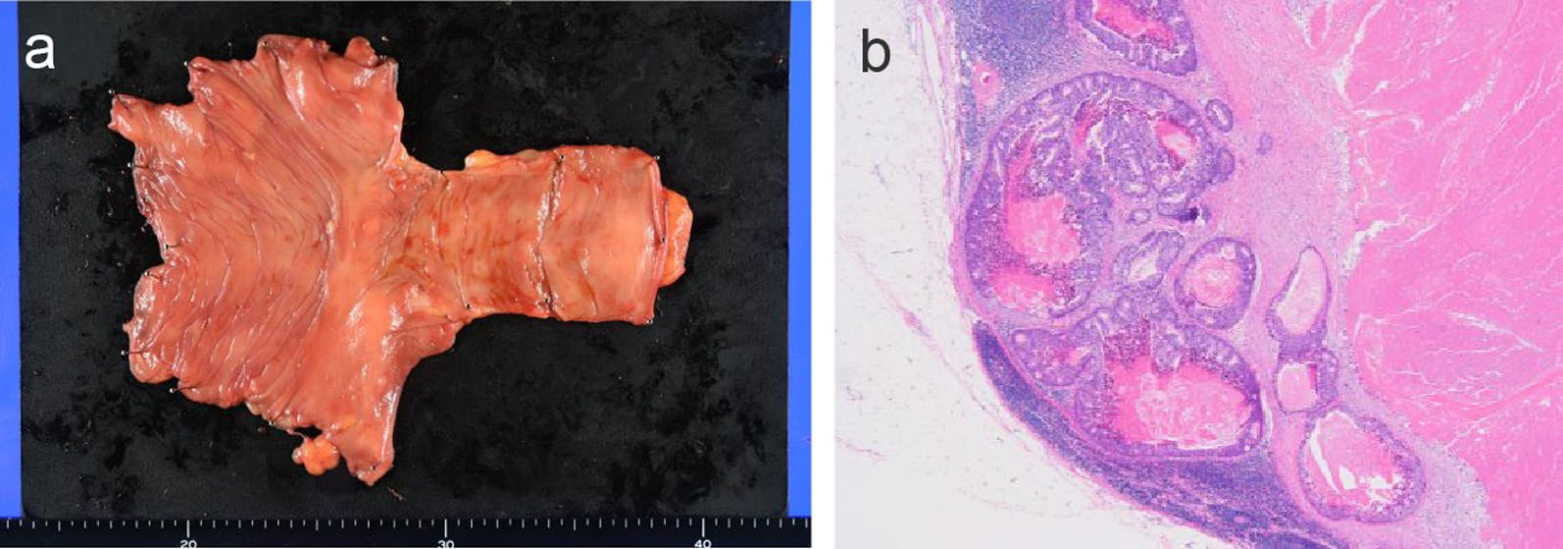
**a** The resected specimen of the ileocecal area. No neoplastic lesion can be seen on the specimen. **b** Pathological image of the resected ileal lymph node shows amoderately differentiated tubular adenocarcinoma (hematoxylin & eosin staining, × 40)

A 77-year-old male presented abdominal pain and anorexia and visited a previous hospital. Computed tomography (CT) revealed rectal cancer that invades the small intestine with LN metastasis, believed to drain from the tumor-invading small intestine, and multiple liver metastases. He was diagnosed with small bowel obstruction due to rectal cancer invading the small intestine, and a decompression of the small intestine was performed before surgery using a nasal ileus tube. The patient underwent a high anterior resection with D3 LN resection and partial resection of the small intestine, which was invaded by the tumor (Fig. 1a). However, the enlarged ileal LN was not removed, considered a distant metastasis, and hepatic metastases remained. The pathological diagnosis of the primary tumor was a moderately differentiated tubular adenocarcinoma, pT4b (ileal invasion), N0 (0/20). The tumor measured 6 cm. The patient was discharged from the previous hospital on postoperative day 9 and referred to our hospital for further treatment of liver and LN metastases. He had a medical history of atrial fibrillation and was administered rivaroxaban. The serum carcinoembryonic antigen (CEA) level was 17.3 μg/ml (normal range: < 5.0 μg/ml), and the serum carbohydrate antigen 19–9 (CA19-9) was < 2.0 U/ml (normal range: < 37 U/ml). No mutation was found in the RAS and BRAF genes and microsatellite instability status was stable. He underwent 17 cycles of 5-fluorouracil (5-FU), leucovorin, oxaliplatin (mFOLFOX6 regimen), and bevacizumab chemotherapy, which resulted in a partial reduction in the size of the lesions (Fig. 2) and normalization of CA 19–9 value. Hematological tests before hepatic and lymph node resection was shown in Table 1. Therefore, 9 months after the first visit, an open ileocecal resection with ileal mesentery resection was performed for regional LN removal, which secondarily metastasized from the primary rectal cancer, and multiple partial liver resections were performed simultaneously (Fig. 1b). Because the enlarged lymph node was located along the ileocecal artery, the lymph nodes were systematically removed up to the root of the ileocecal artery, equivalent to D2 dissection for colon cancer. There were seven lesions in the liver segments S1 (two lesions), S3, S4/8 (two lesions), and S7 (two lesions). Histopathological analysis did not show a neoplastic lesion in the resected remnant ileum, but there was a moderately differentiated tubular adenocarcinoma in two out of eight regional LNs of the ileum (Fig. 3). In all four resected liver specimens, seven viable metastatic adenocarcinomas remained. These findings were consistent with metastasis from primary rectal cancer. The postoperative course was uneventful, and the patient was discharged on postoperative day 18. The patient underwent a follow-up study, and two bilateral lung metastases were found on a follow-up CT scan 5 months after ileocecal resection with LN removal and liver surgery. The two lung metastases were removed in two lung resections, each on the left and right sides, at an interval of 2 months. The pathological findings were consistent with those of rectal cancer metastases. Multiple liver recurrences were observed at the time of the second lung resection. He underwent four cycles of 5-FU, leucovorin, irinotecan (FOLFIRI), and panitumumab, leading to a partial reduction in the size of the hepatic lesions. Subsequently, a posterior sectionectomy of the liver and partial resection were performed for liver metastasis. All lesions were completely resected; however, multiple metastases to the liver, hepatic hilar LNs, and peritoneal seeding were found 3 months after the second hepatectomy. Chemotherapy with 5-FU, leucovorin, irinotecan, and panitumumab was initiated. He is still alive 39 months after his first visit to our hospital and 40 months after his first surgery for the primary lesion at the previous hospital.

**Table 1:** Laboratory data on admission

Data	Value	Range	Data	Value	Range
Alb (g/dL)	3.6	4.1–5.1	WBC (10^3^/μL)	7.61	3.3–8.6
T-Bil (mg/dL)	0.7	0.4–1.5	Hb (g/dL)	10.3	13.7–16.8
AST (U/L)	27	13–30	Plt (10^4^/μL)	32.6	15.8–34.8
ALT (U/L)	25	10–42	PT-A (%)	96	70–140
BUN (mg/dL)	14.5	8–20	PT-INR	1.02	0.80–1.20
Cre (mg/dL)	0.91	0.65–1.07	APTT (sec)	35	23–40
Na (mEq/L)	141	138–145	CEA (ng/ml)	3.5	0–5.0
K (mEq/L)	4.4	3.6–4.8	CA19-9 (U/ml)	< 2.0	0–37.0
CRP (mg/dL)	0.61	0–0.14			

Alb, albumin; ALT, alanine transaminase; APTT, activated partial thromboplastin time; AST, aspartate aminotransferase; BUN, blood urea nitrogen; CA19-9, carbohydrate antigen 19–9; CEA, carcinoembryonic antigen; Cre, creatinine; CRP, C-reactive protein; eGFR, estimated glomerular filtration rate; Hb, hemoglobin; K, potassium; Na, natrium; Plt, platelets; PT-A, prothrombin activity; PT-INR, prothrombin time international normalized ratio; T-Bil, total bilirubin; WBC, white blood cell count

## Discussion

“Secondary LN metastasis” is defined as metastasis that occurs in the regional LNs of adjacent organs invaded by the primary lesion. Direct small bowel invasion of colorectal cancer is reported to occur in approximately 2.2% of cases [1, 2], but cases of secondary LN metastases are rare. Ueno et al. analyzed 997 resection cases of colorectal cancer and reported direct invasion and secondary LN metastasis frequencies in colorectal cancer [1]. According to their report, 783 of the 997 patients underwent curative resection. Direct tumor invasion into adjacent organs was observed in 90 patients. There were 22 cases of direct small bowel invasion and three secondary regional LN metastases of the invaded small intestine, equivalent to 13.6% of colorectal cancers with small intestine invasion. The PubMed database was searched on January 20th, 2024, for studies reporting secondary LN metastasis of colorectal cancer. The search words were (“secondary LN metastasis” OR “secondary metastasis”) AND (colon OR rectal OR colorectal) AND cancer. A total of 20 articles were found and two cases of secondary LN metastasis of colorectal cancer were reported in the English literature. We gathered details on five cases, including our case (Table 2) [3, 4]. The characteristics of colorectal cancer that cause secondary LN metastasis remain unclear. Ueno et al. speculated that synchronous secondary small mesenteric LN metastases occur when tumor invasion involves the small intestinal mucous membrane and when a fistula is formed [1]. Eto et al. reported a similar case and assumed that the characteristics of metachronous secondary LN metastases were the following: “invasive colon cancer,” “tumor invasion extended to the small intestine submucosa,” “histology type was not well-differentiated tubular adenocarcinoma,” “maximum tumor diameter of > 5.1 cm,” and “few LN metastases in colon cancer regional LN, although most of them were advanced colon cancer [3].” In our case, the primary tumor invaded the small intestinal mucosa, the histological type was a moderately differentiated tubular adenocarcinoma with a diameter of 6 cm, and there were no metastatic lesions in the regional LNs of the primary lesion. The present case partially matched these characteristics. In our case, primary rectal cancer had already metastasized to the regional LN of the invaded small intestine and liver when the tumor was detected. The patient survived 40 months after the first operation. According to the UICC for International Cancer Control criteria, colorectal cancer with secondary LN metastasis is classified as Stage IV because these LNs are not the regional LNs of the primary lesion. According to Kanemitsu et al., the median overall survival of Stage IV colorectal cancer with non-resectable metastatic lesions is 25.9 months (19.9–31.5) when treated with primary tumor resection followed by chemotherapy [5]. However, to the best of our knowledge, the median overall survival of patients with colorectal cancer invading the small intestine and metastasizing to the regional LNs of the intestine treated with surgical resection is 40 months (Table 2) [3, 4]. This suggests that if regional LNs of the small intestine can be resected, patients with secondary LN metastasis of colorectal cancer may have a longer survival time than those with Stage IV colorectal cancer. A previous study reported that the prognosis for colorectal cancer with multiple organ involvement that can be resected in combination is good [2]. Secondary LN metastasis is a distant metastasis; however, it may be possible to obtain a similar prognosis in cases of concurrent resection. In the present case, metastasis occurred in the LNs of the invaded small intestine. There is also a report of direct gastric invasion by transverse colon cancer and metastasis to regional stomach LNs [6]. Colorectal cancer that directly invades the small intestine and other parts of the gastrointestinal tract should be carefully monitored for signs of metastasis to LNs at the invaded site.

**Table 2:** Summary of reported cases of secondary lymph node metastasis

References	Year	Age/sex	Primary location	Maximum tumor diameter	Differentiation	Metastasis to the regional LN of the primary lesion	Invaded depth of small intestine	Survival^a^
Eto et al. [3]	2021	67/M	Sigmoid	6.5 cm	tub2	No	Submucosa	Alive, 1 year and 9 months
Takiyama et al. [4]	2016	80/F	Could not be determined	3 cm	N/D	Yes	N/D	Dead, 2 year and 7 months
Takiyama et al. [4]	2016	79/M	Sigmoid	10 cm	N/D	No	N/D	Dead, 4 years
Takiyama et al. [4]	2016	76/M	Ascending	9 cm	N/D	No	N/D	Dead, 5 year and 7 months
Our case		77/M	Rectum	6 cm	tub2	No	Mucosa	Alive, 3 years and 4 months

N/D, no data

^a^Duration of survival from the operation of the primary lesion

## Conclusions

We present a case of colorectal cancer directly invading the adjoining small intestine and secondarily metastasizes to the regional LNs. After surgical resection of the metastasized LNs in addition to the primary lesion, the patient had relatively long-term survival. When colorectal cancer invades the small intestine, it is advisable to pay attention to the invaded small intestinal LNs during preoperative and postoperative follow-up imaging.

## Data Availability

Data sharing is not applicable to this article, as no datasets were generated or analyzed during the current study.

## Abbreviations

LN: Lymph node
CT: Computed tomography
CEA: Carcinoembryonic antigen
CA19-9: Carbohydrate antigen 19–9
5-FU: 5-Fluorouracil
